# Cobrotoxin from *Naja naja atra* Venom Ameliorates Adriamycin Nephropathy in Rats

**DOI:** 10.1155/2015/450581

**Published:** 2015-11-11

**Authors:** Shu-Zhi Wang, Yin-li Xu, Qi Zhu, Jian-qun Kou, Zheng-Hong Qin

**Affiliations:** Department of Pharmacology and Laboratory of Aging and Nervous Diseases, Jiangsu Key Laboratory of Translational Research and Therapy for Neuro-Psycho-Diseases, Jiangsu Key Laboratory of Preventive and Translational Medicine for Geriatric Diseases, College of Pharmaceutical Science, Soochow University, Suzhou 215123, China

## Abstract

Chronic kidney disease (CKD) becomes a global health problem with high morbidity and mortality. Adriamycin- (ADR-) induced rodent chronic nephropathy is a classic experimental model of human minimal lesion nephrotic syndrome. The present study investigated the effect of cobrotoxin (CTX) on ADR-induced nephropathy. Rats were given 6 mg/kg ADR once through the tail vein to replicate ADR nephropathy model. CTX was administered to rats daily by placing a fast dissolving CTX membrane strip under the tongue starting from 5 days prior to ADR administration until the end of experiment. The results showed that CTX ameliorated the symptoms of ADR nephropathy syndrome with reduced body weight loss, proteinuria, hypoalbuminemia, dyslipidemia, serum electrolyte imbalance, oxidative stress, renal function abnormities, and kidney pathological lesions. Anti-inflammatory cytokine IL-10 expression was elevated after CTX administration in ADR nephropathy model. CTX inhibited the phosphorylation of I*κ*B-*α* and NF-*κ*B p65 nuclear translocation. Meanwhile, CTX upregulated the protein level of podocyte-specific nephrin and downregulated the level of fibrosis-related TGF-*β*. These findings suggest that CTX may be a potential drug for chronic kidney diseases.

## 1. Introduction

Chronic kidney disease (CKD) is recognized as a significant global health problem, owing to its high prevalence and fatality rate [1]. Adriamycin (ADR), a representative of anthracyclines drugs, was thought to be the most effective anticancer medicine ever developed [2], while it was found to induce server adverse effects such as nephrotoxicity and cardiotoxicity in clinic [3, 4]. The ADR-induced nephropathy is characterized by massive proteinuria, hypoalbuminemia, dyslipidemia, edema, and abnormal renal functions [5]. Therefore, ADR-induced chronic nephropathy is the most typical and commonly used experimental model of human minimal lesion nephrotic syndrome [6].

Inflammatory reactions and excessive reactive oxygen species (ROS) production are reported to participate in ADR-induced chronic nephropathy [7, 8]. The activation of NF-*κ*B signaling pathway contributed to proteinuric tubulointerstitial inflammation caused by ADR [9]. Proteinuria, which is a biomarker of renal diseases progression, may be associated with the damage of podocyte structure. Podocyte foot processes acted as the main component of glomerular filtration barrier preventing excessive proteins from leaking out [10, 11]. The consecutive podocyte foot processes are connected via slit diaphragms (SD) [12]. Nephrin protein was identified in SD and participated in maintaining the structure and function of podocytes [13].

Cobra venom from* Naja naja atra* comprises a variety of active peptides such as neurotoxin, cardiotoxin, and phospholipase A2 [14]. Our previous researches showed that* Naja naja atra* venom (NNAV) had beneficial effects on inflammation inhibition [15] and immune regulation [16]. NNAV treatment showed good efficacy in adjuvant arthritis [17] and a murine model of systemic lupus erythematosus [18]. Cobratoxin, a long-chain neurotoxin, from Thailand cobra venom has been demonstrated to have potent actions on inhibiting formalin-induced inflammatory pain [19] and adjuvant arthritis [20]. Cobrotoxin (CTX), a short-chain neurotoxin, was suggested to inhibit NF-*κ*B signaling activation [21] and regulated T lymphocytes [22]. ADR-induced chronic nephropathy is related to inflammation reactions and immune responses. Thus, this study was sought to investigate if CTX had a beneficial effect on ADR-induced nephropathy. Our results proved that NNAV has protective effects on ADR nephropathy [23] and diabetic nephropathy [24]; meanwhile, cardiotoxin purified from NNAV was recently reported to slightly ameliorate ADR nephropathy in rats [25]. Therefore, we conjectured that CTX extracted from NNAV may be useful for ADR nephropathy treatment.

## 2. Materials and Methods

### 2.1. Animals

Male Wistar rats weighting 180–220 g were purchased from the Shanghai SLAC Laboratory Animal Co. Ltd. Rats had free access to food and water and were housed in standard laboratory conditions with temperature 18–22°C, humidity 55 ± 10%, and 12 h light/dark cycle. Animals were acclimatized to the laboratory conditions for at least one week prior to experiments and then randomly assigned to individual groups. Body weight was measured once a week during the entire experimental period. All procedures were approved by the Soochow University Animal Care and Use Committee and were carried out in accordance with the National Institutes of Health Guide for the Care and Use of Laboratory Animals (the National Research Council, 1996) and were proved by the ethical committee of Soochow University.

### 2.2. ADR Induced Nephritic Syndrome

ADR (Adriamycin, doxorubicin hydrochloride) was purchased from Shenzhen Main Luck Pharmaceutics Inc. (Shenzhen, China). ADR-induced nephritic syndrome was produced with a single administration of ADR (6 mg/kg body weight, dissolved in sterile 0.9% saline solution) through the tail vein as previously described [23, 26].

### 2.3. CTX Administration

CTX lyophilized powder was obtained from Orientoxin Biotech Co. Ltd. (Laiyang, Shandong, China). CTX fast dissolving membrane strips were made with a membrane forming apparatus (FA-1000, FUAN, China) in a solution containing hydroxypropyl methylcellulose 1.2 g, hyaluronic acid 0.02 g, glycerol 0.378 g, and double distilled water 20 mL. CTX was administered once a day to rats by placing fast dissolving strip containing CTX (5, 15, and 45 *μ*g/kg) under the tongue.

### 2.4. Measurement of 24 h Urine Protein Output

For urine collection and total urinary protein determination, rats were placed in individual metabolic cages with free access to water and food for 24 h every week during the entire experimental period. Total urinary protein concentration (grams per liter) was determined using the Bradford protein assay kit (Beyotime Institute of Biotechnology, China) and a microplate reader (Infinite M1000 PRO, TECAN, Switzerland).

### 2.5. Blood Serum Biochemical Analysis

On the 42nd day after ADR administration, rats were anesthetized with IP injection of pentobarbital sodium and blood sample were collected from abdominal aorta in centrifuge tubes and left to clot at room temperature for 1 h. Serum was separated by centrifugation at 3000 rpm for 15 min and stored at −80°C and thawed just before use. Serum levels of total protein (TP), albumin (ALB), globulin (GLB), albumin/globulin (ALB/GLB), total cholesterol (CHOL), triglyceride (TG), creatinine (SCr), urea nitrogen (BUN), cystatin C (Cys-C), sodium (Na), chlorine (CL), potassium (K), and phosphorus (P) were measured with commercial available kits and an automatic biochemistry analyzer (Mindray BS-800, Shenzhen, China).

### 2.6. Histological Examination and Immunofluorescence Analysis of Kidney Tissue Samples

Kidneys were removed and immersion-fixed in 4% formalin buffer immediately after rats were killed for histological examinations. After being fixed for 24 h, the kidney samples were embedded in paraffin and laminated to 3 *μ*m slices. Then, the kidney sections were cut with a cryostat and stained with hematoxylin and eosin (HE), periodic acid-Schiff (PAS), and Masson's trichrome, respectively. Morphological and histological observations were performed under an Olympus light microscopy (Olympus, Tokyo, Japan).

Paraffin kidney sections (3 *μ*m) mentioned above were deparaffinized in xylenes and rehydrated in decreasing grades of ethanol and double distilled water. Kidney sections were then permeabilized and blocked in PBS buffer containing 0.5% Triton X-100 and 1% bovine serum albumin for 60 min at room temperature, followed by incubation with primary mouse monoclonal anti-NF-*κ*B p65 antibody (Cell Signaling Technology, MA, USA) overnight at 4°C. After being rinsed three times with PBS, kidney sections were incubated with Alexa Fluor 555-congregated goat anti-mouse IgG (Life Technology, MA, USA) for 2 h at room temperature. Nuclei were counter-stained with 4′,6-diamidino-2-phenylindole (DAPI) for ten minutes. Then, all sections were cover-slipped with Fluoromount aqueous mounting medium (Sigma-Aldrich, Saint Louis, USA) and observed with a laser-scanning confocal microscopy (Zeiss LSM 710, Carl Zeiss, Germany).

### 2.7. Determination of Oxidative Stress and Cytokine Levels

Kidney tissue samples were homogenized in PBS (pH 7.4) buffer solution on ice using a homogenizer and then centrifuged to obtain the supernatant. The kidney tissue homogenate supernatant and serum were used for determination of SOD activity and MDA level using colorimetric enzyme assay kits (Beyotime Institute of Biotechnology, China) following the manufacture's procedures. The IL-10 level in renal homogenate supernatant was detected using the commercial available ELISA kits (Xinlebio, Shanghai, China).

### 2.8. Western Blot Analysis

Renal cortex was homogenized in tissue lysis solution (150 mM NaCl, 10 mM Tris (pH 7.4), 1% Triton-X100, 1% sodium deoxycholate, 1% sodium dodecyl sulfate, and 5 mM EDTA (pH 7.4)) supplemented with protease inhibitors (Protease Inhibitor Cocktail Tablets, Roche, Mannheim, Germany) and phosphatase inhibitors (Phosphatase Inhibitor Cocktail Tablets, Roche, Mannheim, Germany). Supernatants were obtained after 15 min of centrifugation at 12000 g. The protein concentrations were determined using a BCA kit (Pierce Biotechnology, Waltham, MA, USA). Sodium dodecyl sulfate polyacrylamide gel electrophoresis (SDS-PAGE) was used to separate proteins of interest. After electrophoresis, the proteins were transferred to nitrocellulose membranes, which were then blocked in TBST solution (0.1% Tween 20 in Tris-buffer saline) containing 5% (w/v) dry milk for 60 min at room temperature. The blots were then incubated overnight at 4°C with primary antibody against I*κ*B-*α* (Cell Signaling Technology, MA, USA), P-I*κ*B (Cell Signaling Technology, MA, USA), IL-10 (Abcam, MA, USA), BAX (Santa Cruz Biotechnology, CA, USA), TGF-*β* (Abcam, MA, USA), and nephrin (Santa Cruz Biotechnology, CA, USA), followed by incubation with fluorescence secondary antibodies (Li-COR Biosciences, Lincoln, NE, USA). The signal was detected with Odyssey infrared imager (Li-COR Biosciences, Lincoln, NE, USA) and analyzed with ImageJ Software (W. S. Rasband, ImageJ, NIH, Bethesda, MD, USA). The signal intensity of primary antibody was normalized to that of *β*-actin (Sigma-Aldrich, Saint Louis, USA) or GAPDH (Merck Millipore, Darmstadt, Germany).

### 2.9. Statistical Analysis

SPSS 16.0 was used for statistical analysis. All data were expressed as means ± standard deviation and analyzed using a one-way ANOVA. Post hoc comparisons were performed using the Student-Newman-Keuls multiple comparison test. *P* values less than 0.05 were considered statistically significant.

## 3. Results

### 3.1. CTX Reduced Body Weight Loss in ADR Nephropathy

**Figure 1:**
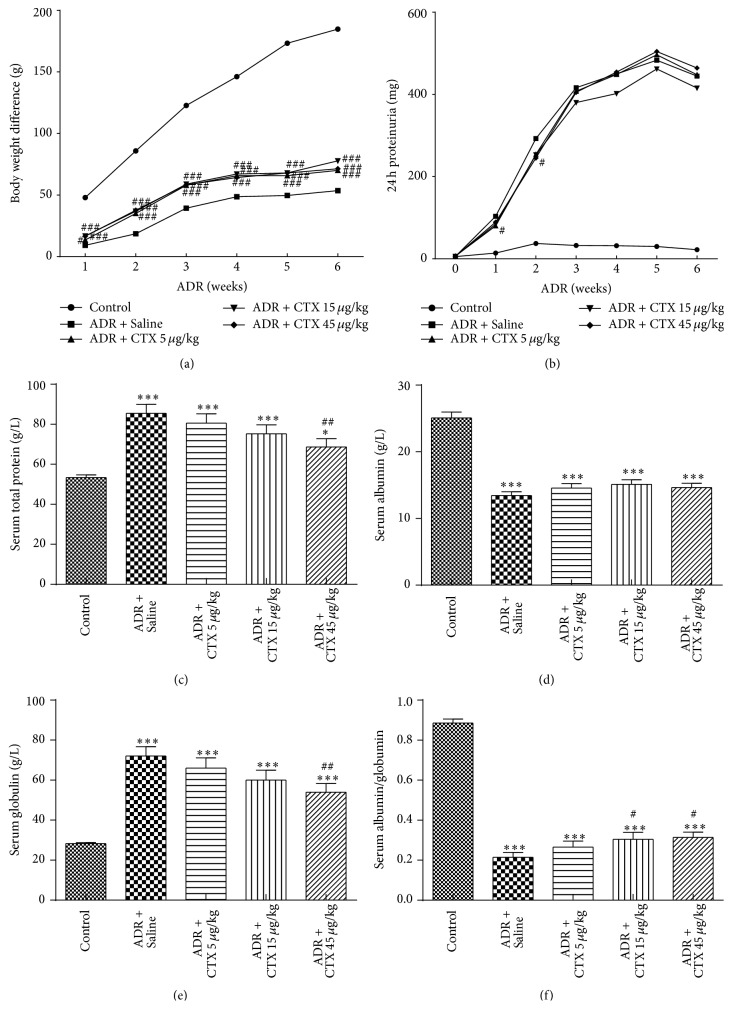
CTX ameliorated body weight loss, 24 h proteinuria excretion, and plasma protein abnormality in ADR nephropathy rats. Adriamycin (ADR, 6 mg/kg) was administered through tail vein injection to induce nephropathy. CTX membrane strips at doses of 5, 15, and 45 *μ*g/kg were administrated to Wistar rats once a day under the tongue 5 days before ADR and continued to the end of experiment. Body weight was measured once a week during the entire experimental period after ADR administration (a). Urine was collected for determination of 24 h proteinuria once a week before and after ADR injection (b). Rats were killed at 6th week and blood samples were collected for determination of serum levels of total protein (c), albumin (d), globulin (e), and albumin/globulin ratio (f). ^*∗*^ *P* < 0.05 and ^*∗∗∗*^ *P* < 0.001 compared with “Control” group; ^#^ *P* < 0.05, ^##^ *P* < 0.01, and ^###^ *P* < 0.001 compared with “ADR + Saline” group, *n* = 21–26.

Body weight was measured once a week during the entire experimental period after ADR injection. As shown in Figure 1(a), the body weight gain was significantly reduced after ADR administration as compared with Control group, and the CTX-treated groups at the dosages of 5, 15, and 45 *μ*g/kg mildly recovered body weight gain (*P* < 0.001) as compared to model group (Adriamycin + Saline group).

### 3.2. Effects of CTX on Proteinuria in ADR Nephropathy

Proteinuria is a clinical indicator of many renal diseases that may be related to podocyte injury.  Figure 1(b) showed that rats acquired severe proteinuria after ADR injection and mean urinary protein excretion was 103.41 ± 50.33 mg/24 h at the first week and rapidly increased to 483.15 ± 288.96 mg/24 h at the fifth week after ADR administration in the model group. CTX treatment slightly decreased proteinuria excretion. The mean urinary protein excretion was 80.22 ± 32.47 mg/24 h on day 7 after ADR injection in CTX group administrated with 5 *μ*g/kg CTX (*P* < 0.05, versus model group), and the mean output proteinuria was 245.19 ± 82.42 mg/24 h on day 14 after ADR administration in rats treated with 45 *μ*g/kg CTX (*P* < 0.05, versus model group). These data suggest that CTX may attenuate ADR-induced proteinuria.

### 3.3. CTX Reduced ADR-Trigged Abnormalities in Plasma Protein and Lipid

In the present study, we found that ADR triggered hypoalbuminemia and high level of serum globulin. The lower serum albumin may be related to severe proteinuria excretion, and higher serum globulin level may indicate the body inflammation condition. As showed in Figures 1(c)–1(f), CTX had no significant effect on ADR-induced decline in serum albumin level but significantly decreased serum levels of globulin from 72.04 ± 22.73 g/L (model group) to 66.01 ± 24.52 g/L (CTX 5 *μ*g/kg treated group), 60.06 ± 23.37 g/L (CTX 15 *μ*g/kg treated group), and 53.98 ± 21.97 g/L (CTX 45 *μ*g/kg treated group, *P* < 0.01, versus model group), respectively. Therefore, the levels of serum total protein decreased and serum albumin/globulin ratio increased in CTX-treated rats (*P* < 0.05, versus model group rats).

**Figure 2:**
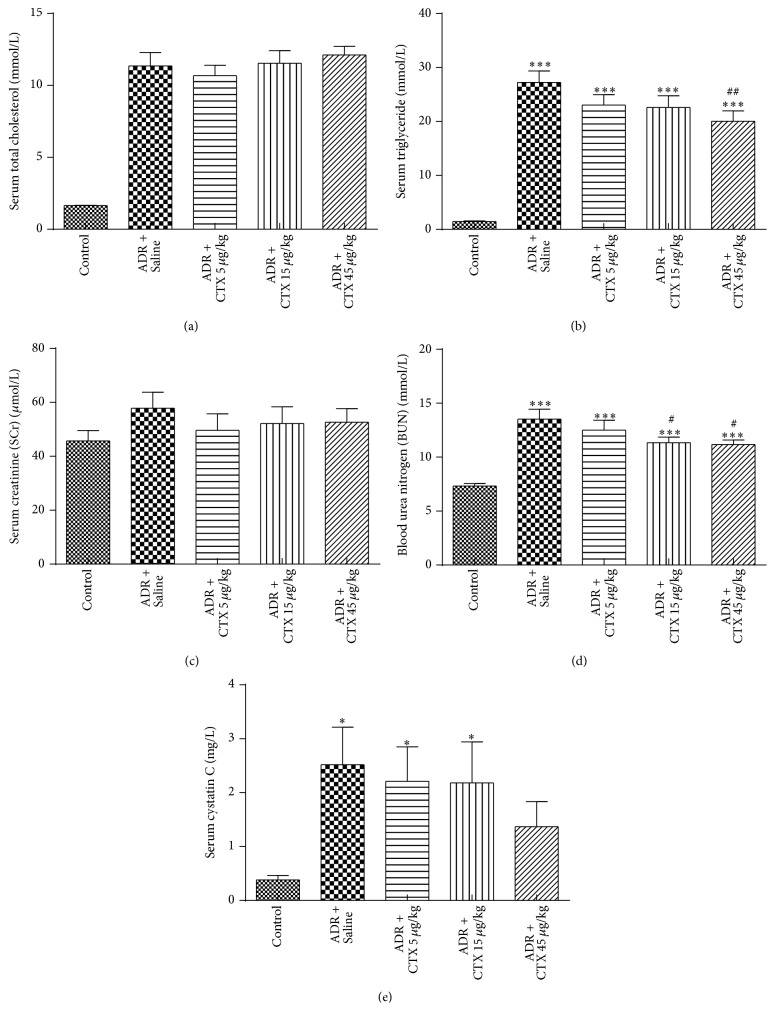
The effects of CTX on hyperlipidaemia and renal function in ADR nephropathy rats. Rats were treated as described in caption of Figure 1. Rats were killed at 6th week and blood samples were collected for determination of serum levels of total cholesterol (a), triglyceride (b), creatinine (c), urea nitrogen (d), and cystatin c (e). ^*∗*^ *P* < 0.05 and ^*∗∗∗*^ *P* < 0.001 compared with “Control” group; ^#^ *P* < 0.05 and ^##^ *P* < 0.01 compared with “ADR + Saline” group, *n* = 21–26.

Hyperlipidaemia is a classic clinical index of nephrotic syndrome and is regarded as a severe risk factor for proteinuria and cardiovascular disease. The present data demonstrated that ADR administration resulted in higher levels of serum total cholesterol and triglyceride. Although there was no significant difference between CTX-treated rats and model group rats in serum total cholesterol level (Figure 2(a)), the CTX therapeutic groups obtained marked lower levels of serum triglyceride (Figure 2(b)), especially in the dose of 45 *μ*g/kg (20.04 ± 9.89 mmol/L, *P* < 0.01) as compared with model group (27.26 ± 10.49 mmol/L).

### 3.4. CTX Improved Kidney Functions

Serum creatinine (SCr) and blood urea nitrogen (BUN) are two major indicators of renal function variables. The current data showed that SCr and BUN levels were increased in rats treated with ADR as compared with normal group (Figures 2(c)-2(d)). CTX therapeutic groups slightly downregulated the SCr level in comparison with model group; however, the BUN level in CTX-treated rats significantly dropped from 13.52 ± 4.52 mmol/L (model group) to 12.51 ± 4.36 mmol/L (5 *μ*g/kg), 11.33 ± 2.48 mmol/L (15 *μ*g/kg, *P* < 0.05, versus model group), and 11.17 ± 2.12 mmol/L (45 *μ*g/kg, *P* < 0.05, versus model group), respectively.

Serum level of cystatin c (Cys-c) is reported to represent the glomerular filtration rate, and it is a more reliable biomarker of renal function. Our present study found a significant increase in serum Cys-c level after ADR injection. CTX tended to lower serum Cys-c levels (Figure 2(e)), but these changes were statistically insignificant due to relative large variations between rats.

**Figure 3:**
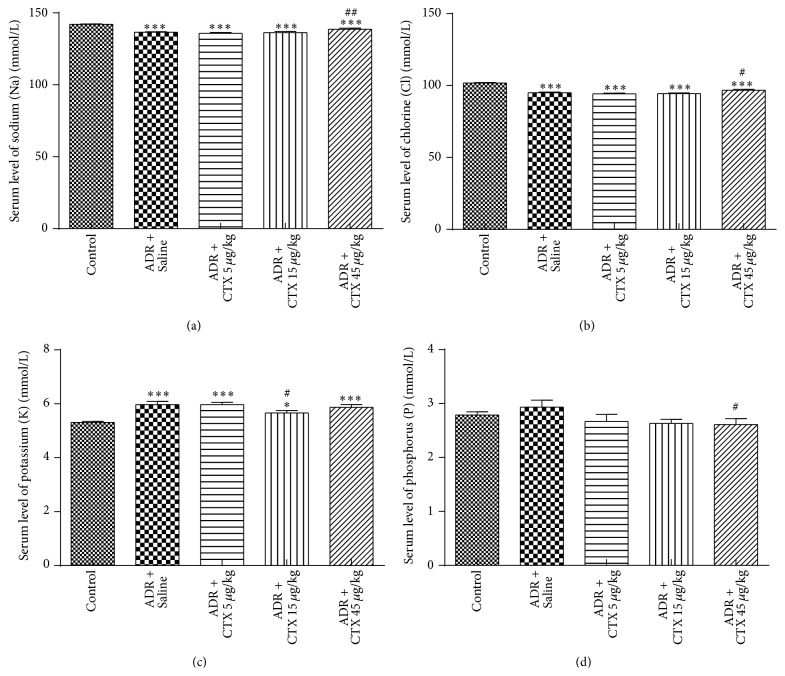
CTX ameliorated imbalance of serum electrolytes in ADR nephropathy rats. Rats were treated as described in caption of Figure 1. Rats were killed at 6th week and blood samples were collected for determination of serum levels of sodium (a), chlorine (b), potassium (c), and phosphorus (d) in ADR model. ^*∗*^ *P* < 0.05 and ^*∗∗∗*^ *P* < 0.001 compared with “Control” group; ^#^ *P* < 0.05 and ^##^ *P* < 0.01 compared with “ADR + Saline” group, *n* = 10–13.

As previous study reported, ADR caused tubular reabsorption dysfunction which led to loss of sodium and chlorine together with retention of potassium and phosphorus [27, 28]. Our present research showed that serum level of sodium and chlorine slightly decreased and serum levels of potassium and phosphorus increased in ADR-administrated rats as compared with normal group (Figures 3(a)–3(d)). These changes were mildly corrected by CTX. CTX at the dose of 45 *μ*g/kg increased serum levels of sodium and chlorine (*P* < 0.05, versus model group). CTX at the dose of 15 *μ*g/kg also reduced serum potassium level from 5.97 ± 0.43 mmol/L (model rats) to 5.67 ± 0.32 mmol/L (*P* < 0.05, compared with model group). Meanwhile, CTX-treated groups obtained slightly lower serum phosphorus level compared with model rats, especially in the dosage of 45 *μ*g/kg (*P* < 0.05, versus model rats). These results demonstrate that CTX may decrease the damage of kidney tubules and maintain renal function.

### 3.5. CTX Reduced ADR-Induced Oxidative Stress

**Figure 4:**
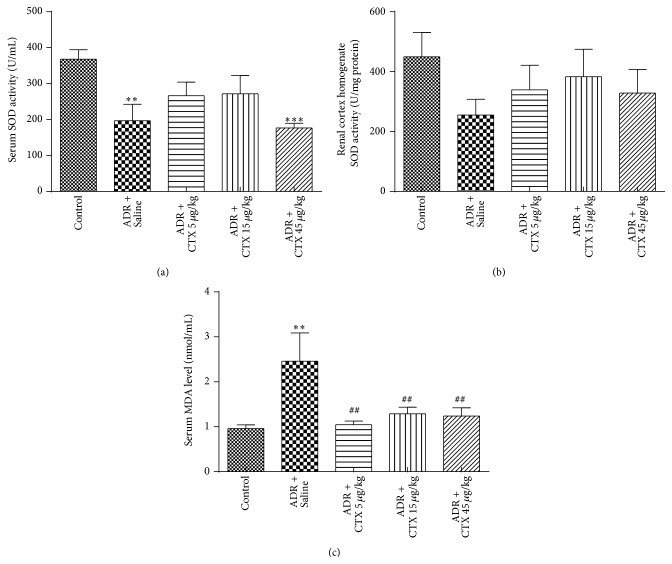
The effects of CTX on oxidative stress in ADR nephropathy rats. Rats were treated as described in caption of Figure 1. Rats were killed at 6th week and blood samples were collected for determination of serum levels of superoxide dismutase (a) and malondialdehyde (c). Renal cortical tissues were dissected for determination of superoxide dismutase (b). ^*∗∗*^ *P* < 0.01 and ^*∗∗∗*^ *P* < 0.001 compared with “Control” group; ^##^ *P* < 0.01 compared with “ADR + Saline” group, *n* = 10–13.

Superoxide dismutase (SOD) is an important antioxidant defense mechanism that prevents body from damages induced by reactive oxygen species. Malondialdehyde (MDA) is a product of lipid peroxidation and also an indicator of level of body oxidative stress. Decreased level of SOD and increased MDA level were found in ADR-treated rats in comparison with normal group (Figures 4(a)–4(c)). The present data demonstrated that CTX had no significant effect on SOD levels in serum and kidney homogenates, but it downregulated serum level of MDA from 2.46 ± 2.09 nmol/mL (model group) to 1.04 ± 0.29 nmol/mL (CTX 5 *μ*g/kg, *P* < 0.01, versus model group), 1.29 ± 0.49 nmol/mL (CTX 15 *μ*g/kg, *P* < 0.01, versus model group), and 1.23 ± 0.63 nmol/mL (CTX 45 *μ*g/kg, *P* < 0.01, versus model group), respectively. These results suggest that CTX may relieve body oxidative stress condition.

### 3.6. CTX Ameliorated Kidney Pathological Changes

**Figure 5:**
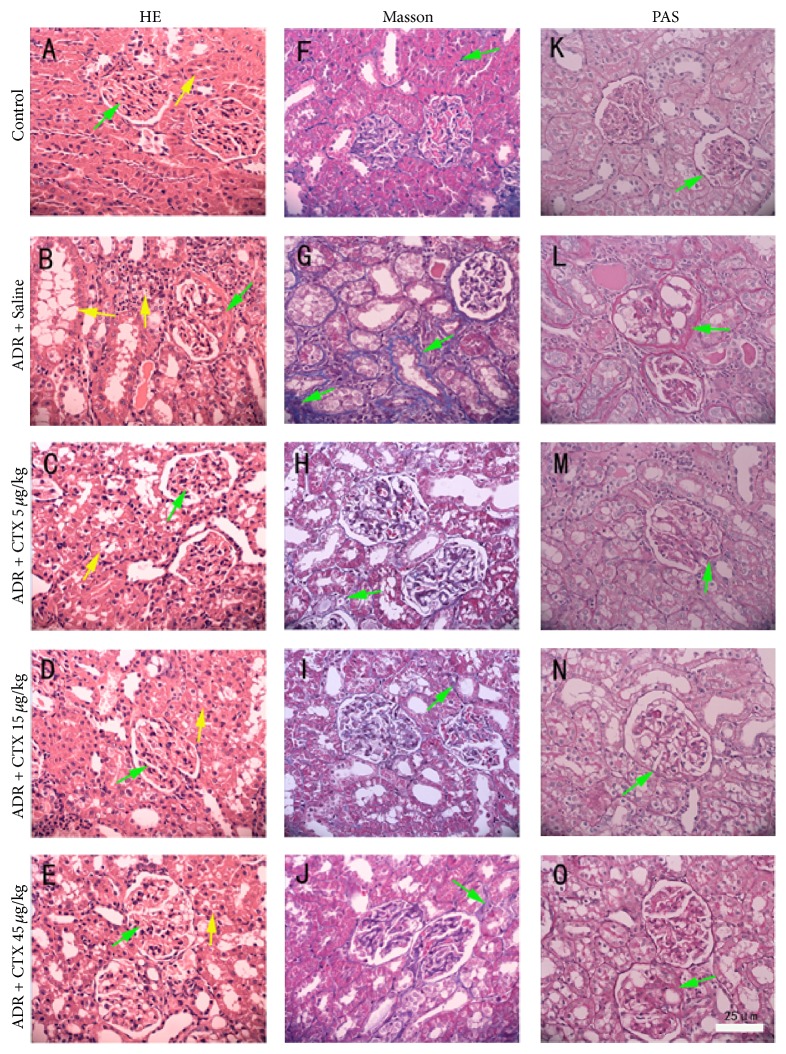
CTX ameliorated kidney pathological changes in ADR nephropathy rats. Rats were treated as described in caption of Figure 1. Rats were killed at 6th week and kidneys were dissected and fixed for hematoxylin and eosin ((A)–(E)), Masson's trichrome ((F)–(J)), and periodic acid-Schiff ((K)–(O)) staining. Kidney histology presented significant pathological lesions characterized by glomerular deformation and damage (green arrow indicated in (B)), tubular epithelial cell vacuolar degeneration and necrosis (yellow arrow indicated in (B)), tubular interstitial collagen proliferation (green arrow showed in (G)), and glomerular basement membrane and mesangial expansion (green arrow showed in (L)) in “ADR + Saline” group rats. CTX ameliorated these pathological changes to varying degrees. Scale bars: 25 *μ*m.

Figure 5 showed the pathological changes of renal histological sections that were stained with hematoxylin and eosin (HE), Masson's trichrome, and Periodic acid-Schiff (PAS), respectively. In HE staining sections (Figures 5(A)–5(E)), Control rats (Figure 5(A)) displayed normal kidney structures, while model group (“Adriamycin + Saline” treated group) presented significant pathological lesions characterized by renal glomerulus capillary congestion, glomerular deformation and atrophy (green arrow indicated in Figure 5(B)), tubular epithelial cell vacuolar degeneration and necrosis, protein cast formation, interstitial edema, and inflammatory cell infiltration (yellow arrows showed in Figure 5(B)). Interestingly, in CTX-treated groups, only slightly renal tubular epithelia cell vacuolar degeneration and inflammatory cell infiltration were observed, and morphological damage of glomerular was lightened (Figures 5(C)–5(E)).

Masson's trichrome staining sections (Figures 5(F)–5(J)) presented tubular congestion and dilation, marked collagen fiber proliferation in tubular interstitial, and fibrosis formation in model group rats (green arrows indicated in Figure 5(G)). However, CTX-treated groups (Figures 5(H)–5(J)) obtained lower extent of tubular collagen proliferation as compared with model group.

PAS staining of kidney tissue sections (Figures 5(K)–5(O)) was mainly purposed to evaluate glomerulosclerosis. Apparently, glomerular basement membrane and mesangial expansion together with mesangial extracellular matrix (ECM) accumulation occurred in model rats (green arrows presented in Figure 5(L)), indicating sclerotic changes. CTX-treated groups (Figures 5(M)–5(O)) showed less mesangial ECM accumulation in glomeruli and largely prevented glomerulosclerosis.

### 3.7. CTX Inhibited Inflammatory Response and Recovered Podocyte-Related Protein Levels

**Figure 6:**
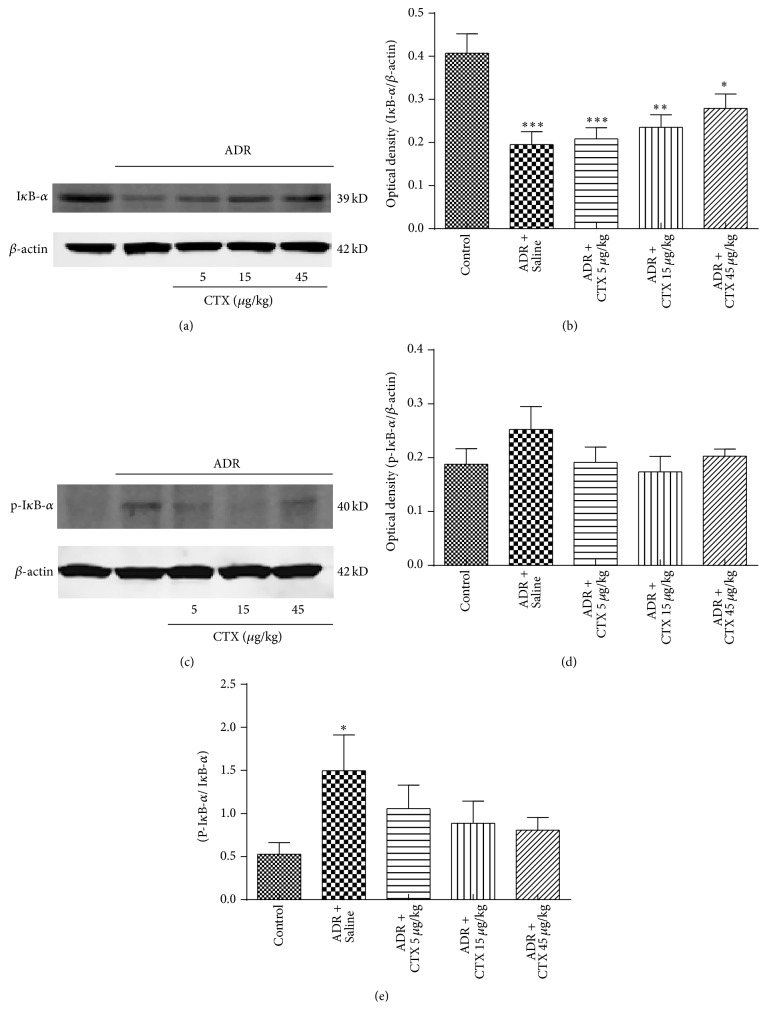
The effects of CTX on the levels of I*κ*B-*α* and p-I*κ*B-*α* in ADR nephropathy rats. Rats were treated as described in caption of Figure 1. Rats were killed at 6th week and I*κ*B-*α* (a) and p-I*κ*B-*α* (c) expressions in renal tissue homogenates were determined with Western blot analysis (*n* = 6). Quantitative analyses of I*κ*B-*α* (b) and p-I*κ*B-*α* (d) levels were performed with ImageJ software and normalized to *β*-actin. Ratio of p-I*κ*B-*α* to I*κ*B-*α* (normalized to *β*-actin resp.) was calculated (e). ^*∗*^ *P* < 0.05, ^*∗∗*^ *P* < 0.01, and ^*∗∗∗*^ *P* < 0.001 compared with “Control” group.

Phosphorylation and degradation of I*κ*B-*α* indicate the activation of NF-*κ*B signaling pathway. As shown in Figure 6, kidney tissue had lower level of I*κ*B-*α* and higher level of p-I*κ*B-*α* expression in model rats compared with Control group, while CTX partially recovered I*κ*B-*α* levels. CTX slightly decreased the level of p-I*κ*B-*α*, but the effect was statistically insignificant due to large variations between rats. Meanwhile, we measured the ratio of p-I*κ*B-*α* to I*κ*B-*α* (normalized to *β*-actin, resp.).  Figure 6(e) demonstrated that model rats obtained higher ratio of p-I*κ*B-*α* to I*κ*B-*α* versus Control group (*P* < 0.05). CTX treatment decreased the ratio of p-I*κ*B-*α* to I*κ*B-*α*.

**Figure 7:**
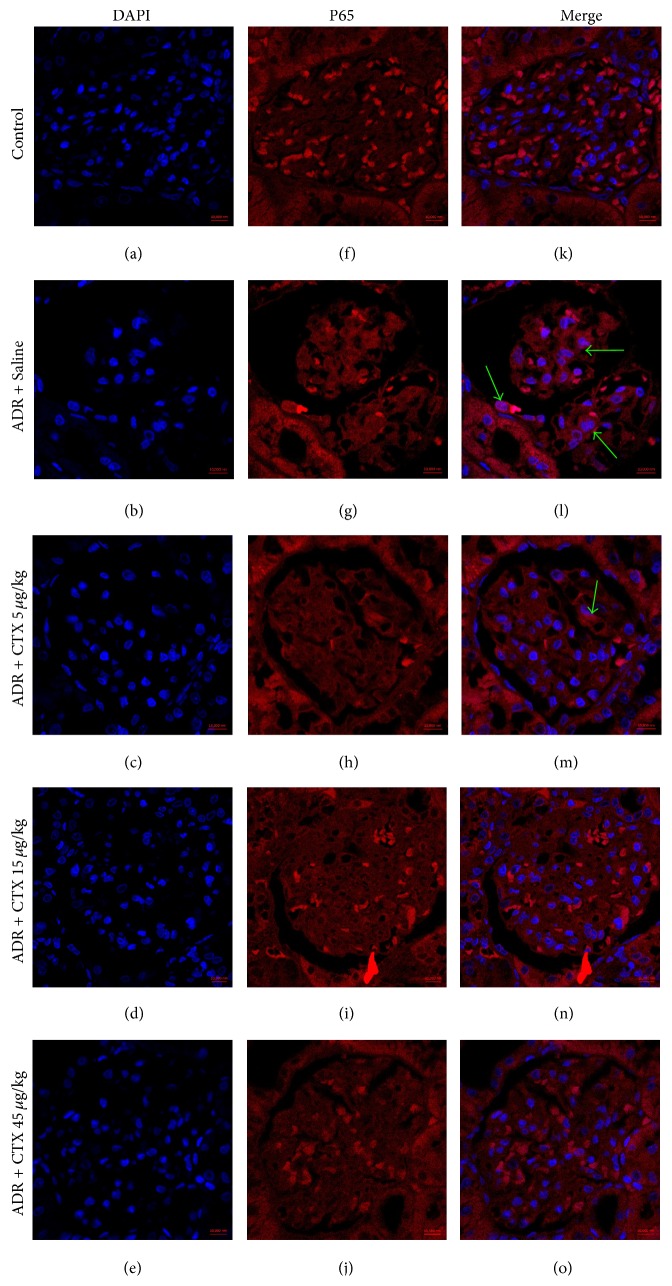
The effects of CTX on NF-*κ*B p65 activation in ADR nephropathy rats. Rats were treated as described in caption of Figure 1. Rats were killed at 6th week and nuclear translocation of NF-*κ*B p65 was determined with kidney paraffin sections by immunofluorescence analysis. The nuclei were stained with DAPI ((a)–(e)) and NF-*κ*B p65 was stained with Alexa Fluor 555 goat anti-mouse IgG ((f)–(j)). Overlay of the images ((k)–(o)) indicated the nuclear translocation of NF-*κ*B p65 (green arrows). Scale bars: 10 *μ*m.

Immunofluorescence analysis (Figure 7) proved the upregulation of NF-*κ*B p65 expression in cytoplasm, together with increased p65 translocation to the nuclei (green arrows indicated in Figure 7) in part of glomerular and tubular cells after ADR administration. However, CTX treatment reduced p65 nuclear translocation compared with model rats.

**Figure 8:**
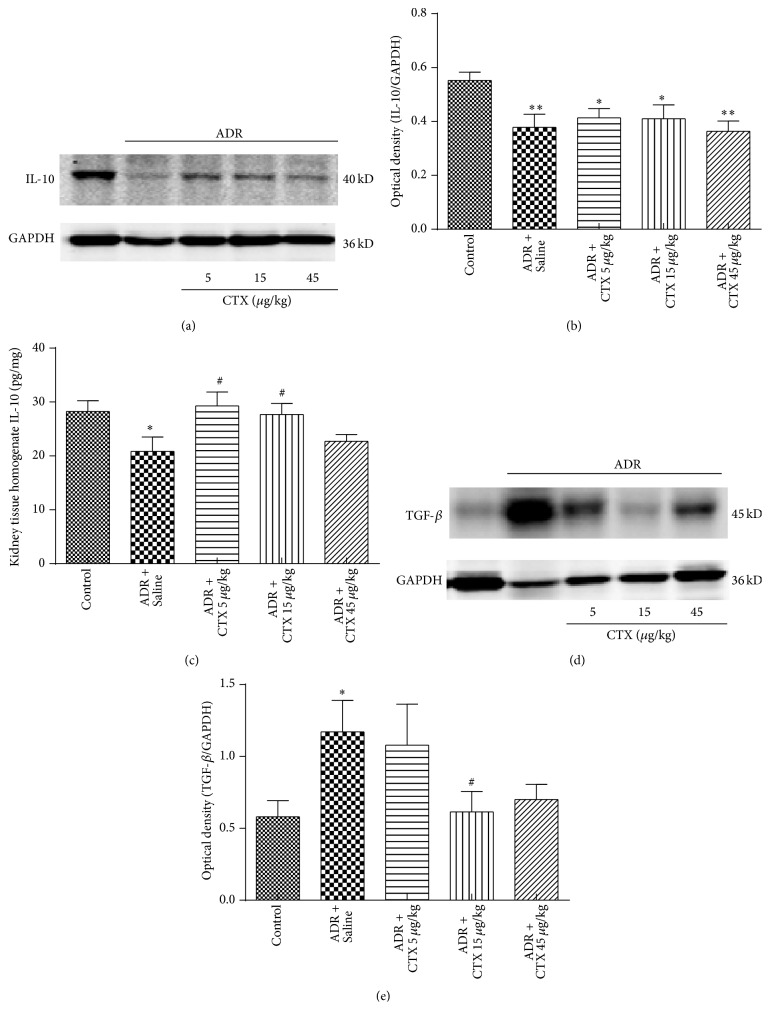
The effects of CTX on the levels of IL-10 and TGF-*β* in ADR nephropathy rats. Rats were treated as described in caption of Figure 1. Rats were killed at 6th week and IL-10 (a) and TGF-*β* (d) expressions in renal tissue homogenates were determined with Western blot analysis (*n* = 6). Quantitative analyses of IL-10 (b) and TGF-*β* (e) levels were performed with ImageJ software and normalized to GAPDH. IL-10 level in kidney tissue homogenates (c) was determined using ELISA kits. ^*∗*^ *P* < 0.05 and ^*∗∗*^ *P* < 0.01 compared with “Control” group. ^#^ *P* < 0.05 compared with “ADR + Saline” group.

Interleukin-10 (IL-10) is an important anti-inflammatory cytokine that may regulate body inflammatory and immune system. Our results suggested that levels of IL-10 in renal tissues, analyzed with Western blotting and Elisa assays (Figures 8(a)–8(c)), were downregulated after ADR administration. Interestingly, CTX treatment elevated kidney tissue IL-10 levels, indicating that CTX may improve inflammatory response.

Transforming growth factor- (TGF-) *β* plays a considerable role in progression of renal fibrosis. Western blot analysis (Figures 8(d)-8(e)) revealed that renal level of TGF-*β* was significantly upregulated in model rats as compared with Control group (*P* < 0.05), while CTX downregulated kidney TGF-*β* level, especially at the dose of 15 *μ*g/kg group (*P* < 0.05, versus model rats).

**Figure 9:**
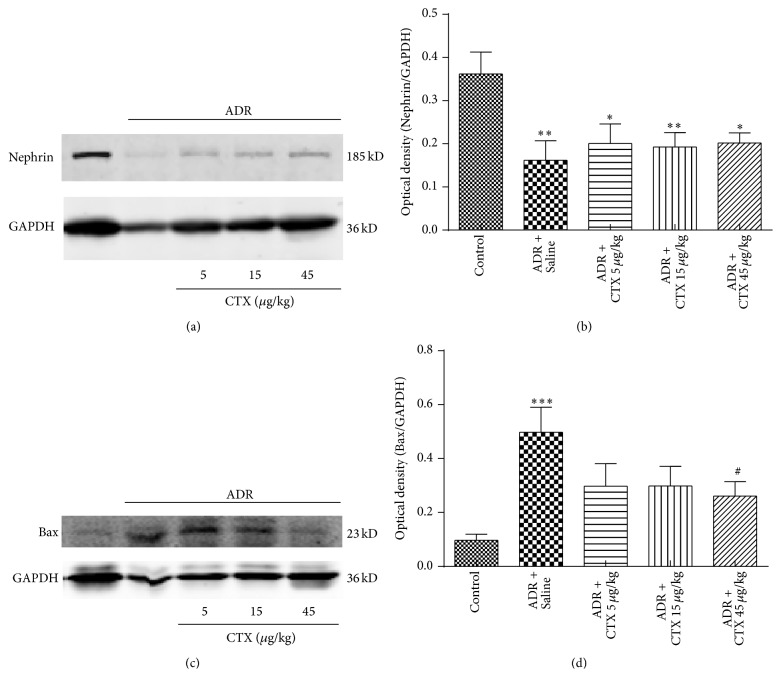
The effects of CTX on the levels of nephrin and Bax in ADR nephropathy rats. Rats were treated as described in caption of Figure 1. Rats were killed at 6th week and nephrin (a) and Bax (c) expressions in renal tissue homogenates were determined with Western blot analysis (*n* = 6). Quantitative analyses of nephrin (b) and Bax (d) levels were performed with ImageJ software and normalized to GAPDH. ^*∗*^ *P* < 0.05, ^*∗∗*^ *P* < 0.01, and ^*∗∗∗*^ *P* < 0.001 compared with “Control” group. ^#^ *P* < 0.05 compared with “ADR + Saline” group.

The kidney podocyte is an important part of glomerular filtration barrier. Alteration of kidney nephrin expression is associated with podocyte damage. ADR caused the downregulation of nephrin expression (Figures 9(a)-9(b)); however, rats treated with CTX slightly reversed nephrin expression descent as compared with model group, suggesting that CTX may protect podocyte from injury induced by ADR.

Renal morphology analysis (Figure 5) showed that ADR caused the injury and apoptosis of glomerular and tubular cells, and Bax is a proapoptotic regulator that influences a series of cellular activities. Our results (Figures 9(c)-9(d)) indicated that renal Bax expression was markedly upregulated in model rats in comparison with Control group (*P* < 0.001). Rats supplied with CTX obtained apparently lower renal Bax expression as compared with model group, especially at the dose of 45 *μ*g/kg group (*P* < 0.05).

## 4. Discussion

The present study demonstrated that CTX has multiple beneficial effects on ADR nephropathy in rats. The most common adverse effect of chemotherapeutic drugs was body weight reduction. Our present data showed that treatment with CTX decreased ADR-induced loss of body weight. Nephropathy patients present a rapid decline in renal function together with decrease in glomerular filtration rate (GFR) [29]. GFR referred to the flow rate of kidney filtered fluid, which is considered as the general indicator of renal function. SCr and BUN are commonly used indices of renal function in clinical studies [30, 31]. Serum Cys-C level is not influenced by muscle mass and health status and recently found to be a reliable marker of GFR [32]. The present research demonstrated that CTX-treated rats obtained lower SCr, BUN, and serum Cys-C levels, indicating that CTX may decrease kidney damage and maintain normal renal function.

Proteinuria is attributed to impairment of glomerular filtration barrier. Glomerular filtration barrier is composed of glomerular endothelial cells, glomerular basement membrane, and podocytes. Podocytes, which have foot processes covering the basement membrane, are crucial component for regulation of glomerular permeability [33]. Neighboring podocyte foot processes are connected by slit diaphragms (SD), which is a lipid raft-like structure that contains multiple proteins [34]. Nephrin is now accepted as the essential SD protein that maintains the structure and function of podocytes and glomerular filtration barrier. Previous studies reported that ADR caused severe loss of nephrin protein and proteinuria. In our present study, CTX treatment only slightly recovered the nephrin level; we predicate that CTX may have a minor protective effect on podocytes.

Excessive filtered proteins that presented in kidney tubules provoke tubulointerstitial injury, tubular epithelial cell necrosis, and inflammatory cells infiltration and finally lead to fibrosis formation in kidney. It is well known that transforming growth factor- (TGF-) *β* plays an important role in the progression of renal fibrosis [35]. Myofibroblast activation and epithelial-mesenchymal transition (EMT) were reported to be triggered by TGF-*β* [36]. Bax, a proapoptotic protein, is involved in apoptosis of renal tubular cells and podocytes in ADR nephropathy [37]. In our present study, renal pathological lesions characterized by glomerular desquamation, renal tubules necrosis, tubulointerstitial injury, and collagen overproduction were marked ameliorated in CTX-administrated rats. Meanwhile, renal expression of TGF-*β* and Bax was apparently downregulated after CTX treatment.

Excessive uric protein leakage also caused hypoalbuminemia in ADR-induced nephrotic syndrome. Hypoalbuminemia is associated with underlying inflammation and malnutrition, and it is also a predictor of cardiovascular disease in CKD [38]. The increase in serum globulin level represents body inflammation and immunity condition. Our recent results indicate that CTX treatment slightly relieved hypoalbuminemia condition and decreased serum globulin level in ADR nephrotic rats, suggesting that CTX may be beneficial for nephropathy restitution. Hypoalbuminemia induces marked albumin and lipoprotein synthesis. However, abnormalities of lipid metabolism may lead to hyperlipidaemia in nephrotic syndrome [39]. Hyperlipidaemia, which is the major risk factor for cardiovascular disease, myocardial infarction, and stroke, may aggravate the progression of renal disease [40]. In the present study, CTX-treated rats obtained lower serum levels of triglyceride in ADR rodent model.

Abnormalities of serum electrolytes are correlated with renal function alteration. ADR triggered proteinuria and dysfunction of tubular reabsorption that induced superabundant sodium and chlorine excretion. Descent of serum sodium concentration may increase the risk of mortality of CKD [41]. Serum potassium and phosphorus levels are apparently elevated in patients with the end-stage renal disease [42]. Hyperkalemia may provoke serious electrocardiographic abnormalities and cardiovascular diseases [43]. Hyperphosphatemia becomes a well-recognized complication of end-stage renal disease. Phenotypic changes of vascular smooth-muscle cells and vascular calcification are relevant to exorbitant serum phosphorus level, which is also a risk factor for cardiovascular mortality in CKD [44, 45]. Our current results demonstrated that CTX treatment ameliorated serum electrolytes dysregulation by sustaining serum sodium and chlorine levels and improving hyperkalemia and hyperphosphatemia conditions. However, the magnitudes of changes in electrolytes in model group and in groups receiving CTX were small, and thus the clinical significance of the effects of CTX on electrolytes remains uncertain.

Oxidative stress plays important roles in the development and progression of renal diseases. Increased reactive oxygen species (ROS) and superoxide production may aggravate renal impairments via cellular apoptosis, DNA damages, and lipid peroxidation [46, 47]. Superoxide dismutase (SOD) enzyme catalyzes the dismutation of superoxide radical into oxygen and hydrogen peroxide. SOD is accepted as a potent antioxidant that prevents body from ROS and oxidative stress injuries [48]. Malondialdehyde (MDA) is the most toxic product of lipid peroxidation, which triggers excessive oxidative damages and cells apoptosis or necrosis [49]. Our current studies showed that CTX may reduce oxidative stress as evidenced by decreasing MDA generation in ADR murine nephropathy model.

NF-*κ*B is a transcriptional factor which modulates cellular stress reactions, inflammation, and immune responses [50]. Under normal conditions, NF-*κ*B binds to inhibitory I*κ*B proteins (such as I*κ*B-*α*) and exists as a complex in the cytoplasm. Phosphorylation and degradation of I*κ*B-*α* in various pathological situations would provoke the translocation of disengaged NF-*κ*B to the nucleus and activate transcription of target genes, which promotes inflammatory and immune reactions [51]. Our present study found that CTX treatment increased I*κ*B-*α* levels, slightly downregulated p-I*κ*B-*α* levels, and inhibited NF-*κ*B nuclear localization in ADR nephropathy. IL-10, a potent anti-inflammatory cytokine, was reported to inhibit inflammation, glomerulosclerosis progression, and interstitial fibrosis and improve renal function in CKD [52]. Rats in CTX treatment group obtained higher IL-10 expression than that in model group in our current research, indicating that CTX may inhibit inflammatory reactions in ADR rodent models.

## 5. Conclusions

We demonstrated that CTX ameliorated ADR-induced symptoms of nephrotic syndrome via improving proteinuria excretion, hypoalbuminemia, and dyslipidemia and reducing oxidative stress reactions and renal pathological impairments. Meanwhile, CTX inhibited NF-*κ*B mediated inflammation and TGF-*β* related tubular interstitial fibrosis formation. The present findings suggest potential usefulness of CTX in chronic nephropathy.
